# Second-Tier Whole Exome Sequencing Following Abnormal Newborn Screening: Diagnostic Yield, Secondary Findings, and Carrier Burden in a Taiwanese Neonatal Cohort

**DOI:** 10.3390/children13080977

**Published:** 2026-07-23

**Authors:** Cheng-Yu Lee, Dau-Ming Niu, Chia-Feng Yang, Yann-Jang Chen

**Affiliations:** 1 Department of Pediatrics, Taipei Veterans General Hospital, Taipei 112, Taiwandmniu1111@yahoo.com.tw (D.-M.N.); cfyang3@vghtpe.gov.tw (C.-F.Y.); 2 Institute of Clinical Medicine, School of Medicine, National Yang Ming Chiao Tung University, Taipei 112, Taiwan; 3 Department of Life Sciences and Institute of Genome Sciences, National Yang Ming Chiao Tung University, Taipei 112, Taiwan; 4 Department of Education and Research, Taipei City Hospital, Taipei 106, Taiwan

**Keywords:** newborn screening, whole exome sequencing, inborn errors of metabolism, secondary findings, carrier burden, East Asian, Taiwan

## Abstract

**Highlights:**

**What are the main findings?**

- In 118 Taiwanese neonates with abnormal biochemical newborn screening, second-tier whole exome sequencing (WES) confirmed a molecular diagnosis in 42.4% and identified carriers in a further 38.1%, reliably separating true disease from carrier, pseudodeficiency, and biochemical false-positive states.
- Beyond the referral indication, P/LP secondary findings (ACMG SF v3.3) were present in 4.2% of neonates, incidental findings in 51.7%, and at least one recessive-carrier variant in 99.2%.

**What are the implications of the main findings?**

- WES is a high-value confirmatory second-tier test that sharpens diagnostic precision in neonatal screening, but its yield depends on population-contextualized interpretation—curated hotspot and pseudodeficiency annotation for variants.
- The near-universal carrier burden and high incidental-finding rate make comprehensive, opt-in pre- and post-test genetic counseling indispensable to responsible genomic newborn screening programs in health systems.

**Abstract:**

**Background/Objectives:** Whole exome sequencing (WES) has emerged as a clinically valuable second-tier test following abnormal biochemical newborn screening (NBS). However, population-specific data on diagnostic yield, secondary findings (SFs), and exome-wide carrier burden remain scarce in East Asian neonates, particularly since the release of the ACMG SF v3.3 gene list. We aimed to characterize these metrics in a Taiwanese neonatal cohort. **Methods:** We retrospectively analyzed 118 consecutive neonates referred to Taipei Veterans General Hospital between August 2021 and August 2022 following abnormal biochemical NBS. WES was performed on the Illumina NovaSeq 6000 platform; variants were classified per the 2015 ACMG/AMP framework and re-evaluated under ACMG SF v3.3. Referral categories comprised lysosomal storage diseases (*n* = 61), amino acid disorders (*n* = 38), fatty acid oxidation disorders (*n* = 13), and organic acid disorders (*n* = 6). **Results:** Fifty neonates (42.4%) received confirmed molecular diagnoses and 45 (38.1%) were carriers (combined molecular resolution 80.5%). Five participants (4.2%) harbored pathogenic/likely pathogenic variants in ACMG SF v3.3 genes (*TTN*, *LDLR*, *PTEN*, *RYR1*, *TP53*). Incidental findings occurred in 51.7%, and at least one recessive-carrier variant was detected in 99.2% (mean 4.15 per individual; median 4). The Taiwanese-specific c.639+919G>A cardiac Fabry variant accounted for 24/25 confirmed male Fabry cases, and the p.Gly576Ser pseudodeficiency allele confounded all suspected Pompe cases. **Conclusions:** Second-tier WES substantially improves diagnostic precision, discriminating confirmed diagnoses from carrier, pseudodeficiency, and biochemical false-positive states. The high carrier burden and incidental-finding rate underscore the importance of comprehensive pre- and post-test genetic counseling in East Asian neonatal genomic programs.

## 1. Introduction

Newborn screening (NBS) represents one of the most cost-effective public health programs in modern medicine, enabling early detection and treatment of life-threatening conditions before symptom onset [1,2]. Conventional NBS relies primarily on biochemical analysis—chiefly tandem mass spectrometry (MS/MS)—to identify abnormal metabolite profiles in dried blood spots. While highly effective, biochemical screening carries well-recognized limitations: analyte overlap between disorders, dietary and maternal confounders, pseudodeficiency alleles, and variant-specific false positives, all of which can cause parental anxiety, unnecessary investigations, and delayed diagnoses [3,4]. In Taiwan, these limitations are particularly pronounced for Pompe disease (*GAA* p.Gly576Ser pseudodeficiency) and Fabry disease (cardiac-variant *GLA* c.639+919G>A) [5,6].

Next-generation sequencing (NGS), and in particular whole exome sequencing (WES), has created new opportunities to complement biochemical NBS as sequencing costs and turnaround times have decreased [7]. Major international genomic NBS initiatives—BabySeq, NBSeq, and NC NEXUS—have established proof-of-concept for WES in the neonatal context [8,9,10]. More recently, the Australian BabyScreen+ prospective study demonstrated that whole-genome sequencing of 1000 neonates was feasible and clinically impactful, identifying high-chance results in 1.6% of infants—of whom only one was detected by standard biochemical NBS [11]. The U.S. Early Check program reported a 2.5% screen-positive rate with 55% confirmed as true positives across ~2000 newborns [12], and the GUARDIAN study identified clinically important conditions in 3.0% of 4000 New York newborns, 90% of which would have been missed by conventional NBS [13]. The UK Generation Study has further extended this framework to 100,000 infants [14].

Despite these advances, genomic sequencing alone remains insufficient as a first-tier NBS tool. The NBSeq study demonstrated that WES achieves a sensitivity of 88% and specificity of 98.4% for inborn errors of metabolism (IEMs)—inferior to MS/MS (99.0% and 99.8%) [9]. A Chinese multicenter study of 29,601 newborns confirmed that combining an NGS panel with MS/MS achieves superior diagnostic performance over either modality alone [15]. Consequently, a tiered model—biochemistry first, sequencing second—has consolidated internationally, with WES serving as a confirmatory second-tier test to resolve biochemically abnormal cases, distinguish disease from carrier status, identify pseudodeficiency alleles, and reduce false positives.

Interpreting WES results in newborns presents unique challenges, including incomplete penetrance, variable expressivity, and the limited phenotypic information available in the neonatal period. Sequencing also yields findings unrelated to the clinical indication—including secondary findings (SFs) in actionable disease genes and exome-wide incidental findings—the reporting of which is governed by evolving international guidelines. The American College of Medical Genetics and Genomics (ACMG) maintains a minimum list of medically actionable genes, updated annually: ACMG SF v3.2 (81 genes, 2023) incorporated *CALM1–3*, and ACMG SF v3.3 (84 genes, February 2025) adds *ABCD1* (X-linked adrenoleukodystrophy) together with further cardiovascular genes [16,17].

Population-specific allele frequencies, pseudodeficiency variants, and carrier landscapes collectively shape the yield and clinical utility of neonatal WES. Taiwanese and broader East Asian populations harbor distinct spectra for Fabry disease (highly prevalent IVS4+919G>A cardiac variant) [6], Pompe disease (*GAA* pseudodeficiency) [5], and *GJB2*-related hearing loss—features that are under-represented in European-derived reference cohorts [18]. Comprehensive Taiwan-specific datasets on second-tier neonatal WES remain scarce.

Here we report an integrated analysis of WES outcomes in 118 consecutive Taiwanese neonates referred for abnormal biochemical NBS at a tertiary genetic-counseling center. Our prespecified objectives were to: (1) quantify diagnostic yield and carrier detection across major IEM categories; (2) determine the frequency and clinical spectrum of SFs under the updated ACMG SF v3.3 gene list; (3) assess the prevalence of exome-wide incidental findings; and (4) characterize the recessive-carrier landscape of the Taiwanese neonatal exome. This work provides a population-contextualized benchmark to guide the deployment of genomic NBS across East Asian health systems.

## 2. Materials and Methods

### 2.1. Study Design and Participants

This observational retrospective study was conducted at the Department of Pediatrics, Taipei Veterans General Hospital (TVGH), Taipei, Taiwan. Between 5 August 2021 and 15 August 2022, 118 consecutive neonates referred for second-tier WES following abnormal biochemical NBS were enrolled. Eligibility required at least one abnormal primary analyte or enzyme activity detected on MS/MS or a targeted enzyme assay. Exclusion criteria were insufficient DNA yield for library preparation and prior molecular diagnosis by alternative methods. Written informed consent was obtained from parents or legal guardians, and structured pre-test genetic counseling was provided by board-certified genetic counselors in all cases. All families received genetic counseling and could independently opt in or opt out of receiving each category of additional results (secondary findings, incidental findings, and carrier status). Only the categories that a family elected to receive were disclosed, and all disclosed results were returned in the setting of post-test genetic counseling.

### 2.2. Whole Exome Sequencing

Genomic DNA was extracted from peripheral blood. Libraries were prepared using the KAPA HyperPrep Kit (Roche, Basel, Switzerland), exome-enriched with a standard capture platform, and sequenced on the Illumina NovaSeq 6000 (Illumina, San Diego, CA, USA) using 150-bp paired-end reads, targeting a minimum mean coverage of 100×. Reads were aligned to GRCh37/hg19 and processed in CLC Genomics Workbench v12.0 (Qiagen, Hilden, Germany). Bases with Phred < 30 were trimmed, mapping was performed at a similarity threshold of 0.9, and a single best hit per read was retained. Across the cohort, the mean sequencing depth was 184.6 X, with 98.73% of targeted bases covered at ≥20 X. Certain genes showed inadequate coverage by WES. For instance, the *CBS* gene achieved only approximately 30% coverage, whereas the *ASS1* gene typically achieved less than 70% coverage. To ensure complete sequencing of these genes, the regions with insufficient WES coverage were further analyzed by Sanger sequencing. The large pathological insertion mutation of IVS16ins3kb *SLC25A13* was also analyzed by PCR.

### 2.3. Variant Filtering, Annotation, and Orthogonal Confirmation

Variants were filtered against dbSNP v150 and the Taiwan Biobank database to exclude common population variants. Candidate genes were prioritized using phenotype-driven Human Phenotype Ontology (HPO) term lists corresponding to each referral indication. Coding (CDS), 5′/3′-UTR, and canonical splice-site variants were retained; missense calls were further evaluated with SIFT and PROVEAN. Variants classified as benign or likely benign in ClinVar were excluded. Because short-read WES under-detects large deletions, tandem repeats, and structural variants, orthogonal Sanger sequencing was performed for *CBS*, *SLC25A13*, and *PAH*; additional molecular assays (MLPA, long-range PCR) were used when clinically indicated. Variants identified in genes related to the primary referral indication were confirmed by Sanger sequencing before being included in the clinical report. In contrast, ACMG secondary findings (SFs) and other incidental findings detected by WES were initially reported without confirmatory Sanger sequencing. Confirmatory Sanger sequencing was performed only upon the request of the patient or family.

### 2.4. Variant Classification

All variants were classified under the joint ACMG/AMP five-tier framework as pathogenic (P), likely pathogenic (LP), variant of uncertain significance (VUS), likely benign (LB), or benign (B) [19]. Only P and LP variants were considered diagnostic; VUS were excluded from primary statistical outputs given their limited neonatal predictive value. A case was considered diagnostic when a single P/LP variant was identified in a dominant-disease gene, or when two P/LP variants were identified in a recessive gene (presumed biallelic).

### 2.5. Secondary Findings (ACMG SF v3.3) and Incidental Findings

Secondary findings were evaluated by systematic analysis of the ACMG SF v3.3 gene list (84 genes; approved February 2025) [17] after exclusion of genes overlapping the primary NBS indication. Incidental findings were defined as reportable P/LP variants in disease-causing genes unrelated to the referral phenotype and not included in the ACMG SF list. A finding was considered reportable if it was (i) a heterozygous P/LP variant in an autosomal-dominant disease gene, or (ii) two or more P/LP variants in an autosomal-recessive gene. Carrier status was reported for any single P/LP heterozygous variant in an autosomal-recessive gene irrespective of the referral indication. For X-linked recessive conditions, a heterozygous P/LP variant in a female was reported as carrier status, whereas a hemizygous P/LP variant in a male was classified as an affected (diagnostic) finding rather than as a carrier. To summarize the three reporting categories: secondary findings were restricted to P/LP variants in the ACMG SF v3.3 gene list; incidental findings comprised reportable P/LP variants in disease genes that were neither on the ACMG SF list nor related to the referral indication; and carrier status denoted a single heterozygous P/LP variant in an autosomal-recessive gene. Because enrollment (August 2021–August 2022) preceded the publication of ACMG SF v3.3 (2025), all exomes were retrospectively reanalyzed against the v3.3 gene list.

### 2.6. Ethical Approval

This study was approved by the Institutional Review Board of Taipei Veterans General Hospital (TPEVGH IRB No. 2021-06-008A) and conducted in accordance with the Declaration of Helsinki. Written informed consent was obtained from all parents or legal guardians of participants included in the study.

## 3. Results

### 3.1. Study Cohort and Diagnostic Yield

A total of 118 neonates (54 female, 64 male) with biochemically abnormal NBS results were enrolled. The referred disorders comprised lysosomal storage diseases (*n* = 61, 51.7%), amino acid disorders (*n* = 38, 32.2%), fatty acid oxidation disorders (*n* = 13, 11.0%), and organic acid disorders (*n* = 6, 5.1%). Following WES and ACMG/AMP variant classification, participants were categorized as confirmed cases (*n* = 50, 42.4%), carriers (*n* = 45, 38.1%), or unresolved (*n* = 23, 19.5%). The combined molecular resolution (diagnosis + carrier) reached 80.5%. Detailed outcomes by disorder category are summarized in Table 1. WES and targeted Sanger sequencing of genes associated with the referred disorders were performed in parallel. The turnaround time for Sanger sequencing was within 2 weeks, whereas WES required approximately 8 weeks. Therefore, the initiation of treatment was primarily guided by the biochemical findings and the Sanger sequencing results.

### 3.2. Amino Acid Disorders

Of 24 neonates referred for citrullinemia, WES identified 11 citrullinemia type I (*ASS1*) carriers, 13 citrullinemia type II (*SLC25A13*) carriers, and 3 confirmed type II cases; three neonates carried variants conferring both type I and type II carrier status. Because WES with short-read sequencing may miss large deletions within *SLC25A13*, orthogonal Sanger sequencing was performed for all citrullinemia cases; it confirmed the WES findings and did not detect additional *SLC25A13* variants beyond those already identified by WES. Among seven neonates referred for homocystinuria, none harbored a pathogenic *CBS* variant. The biochemical hypermethioninemia observed in these cases was associated with heterozygous *MAT1A* variants, which typically cause benign or mild isolated hypermethioninemia rather than classical homocystinuria; accordingly, no confirmed molecular diagnosis of the referred disorder was established and these cases were classified as unresolved. Seven neonates with elevated phenylalanine were confirmed as phenylketonuria (PKU) with pathogenic *PAH* variants; Sanger sequencing was employed to verify large *PAH* deletions not reliably captured by WES.

### 3.3. Fatty Acid Oxidation Disorders

Among 13 neonates referred for fatty acid oxidation disorders, 8 confirmed cases and 3 carriers were identified. The single CPT II carrier harbored heterozygous variants in both *ACADS* and *ACADVL*, both classified as VUS and therefore excluded from the carrier count. Adrenoleukodystrophy (X-ALD) was confirmed in 2 neonates and 2 were carriers. Because X-ALD is a primary referral category in the Taiwanese NBS program, these findings were reported as primary diagnoses rather than secondary findings, notwithstanding the recent addition of *ABCD1* to the ACMG SF v3.3 list (2025), which reflects the availability of pre-symptomatic disease-modifying therapy—allogeneic hematopoietic stem-cell transplantation for cerebral ALD [20].

### 3.4. Organic Acid Disorders

Elevated C5OH (3-hydroxyisovalerylcarnitine), most commonly attributable to 3-methylcrotonyl-CoA carboxylase (3-MCC) deficiency, was the predominant organic acid referral (*n* = 4). P/LP variants in *MCCC1* were identified in 3 carriers, whereas one confirmed case was compound heterozygous for *MCCC2* variants. Biochemical differentials of HMG-CoA lyase deficiency and multiple carboxylase deficiency (biotinidase deficiency and holocarboxylase synthetase deficiency) were excluded by molecular testing.

### 3.5. Lysosomal Storage Diseases

Lysosomal storage diseases formed the largest diagnostic category (*n* = 61). Among 36 Fabry referrals (25 males, 11 females), only one novel *GLA* variant (c.1286_*7del; p.0?) was identified; the remaining 24/25 confirmed male cases harbored the c.639+919G>A (IVS4+919G>A) cardiac variant, consistent with its high prevalence in the Taiwanese population (~1:875 in males) [21,22]. For Pompe disease (*n* = 25), a total of 18 Pompe carriers were detected. Among them, 16 neonates were carriers of the pseudodeficiency allele alongside a *GAA* P/LP variant; a further 2 carriers harbored a single *GAA* P/LP variant without the pseudodeficiency allele. Among the 6 confirmed cases, the East Asian pseudodeficiency allele c.1726G>A (p.Gly576Ser) was present in 5 individuals (1 homozygous), and 1 non-carrier harbored two pseudodeficiency alleles without a pathogenic variant. Compound heterozygosity was verified by parental testing in all confirmed cases, illustrating the critical role of WES in distinguishing true Pompe disease from pseudodeficiency [23,24,25].

### 3.6. Secondary Findings (ACMG SF v3.2 → v3.3)

P/LP variants in ACMG SF v3.2 genes were identified in 5/118 participants (4.2%), consistent with reported prevalence of 3–4% in genomic sequencing cohorts [8,26,27,28]. Identified variants are detailed in Table 2, spanning *TTN* (dilated cardiomyopathy), *LDLR* (familial hypercholesterolemia), *PTEN* (PTEN hamartoma tumor syndrome), *RYR1* (malignant hyperthermia susceptibility), and *TP53* (Li-Fraumeni syndrome). All five families received post-test genetic counseling and appropriate referrals for surveillance and cascade testing. P/LP variants in *HFE* were identified in 6 neonates; however, none carried the p.Cys282Tyr homozygous genotype required for reporting under ACMG SF, and these were therefore not reported as SFs. Re-evaluation under ACMG SF v3.3 did not reclassify any of these five secondary findings and identified no additional secondary finding in this cohort. Although *ABCD1* was newly added to the v3.3 list, it overlaps a primary NBS referral category (X-ALD) in this cohort and was therefore reported as a primary finding rather than a secondary finding.

### 3.7. Incidental Findings

**Figure 1:**
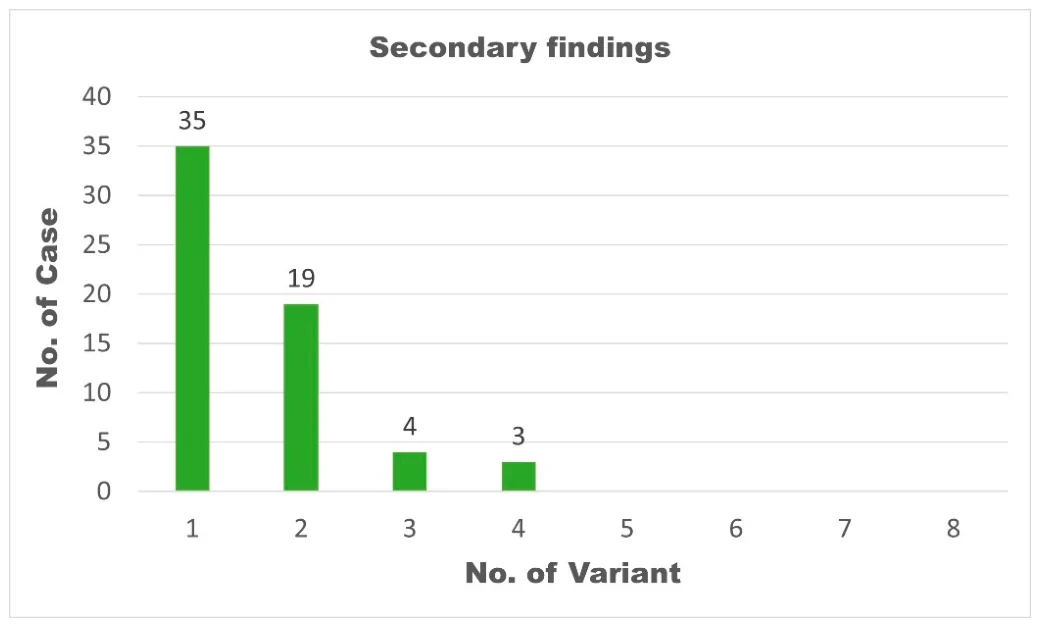
Distribution of incidental findings by number of P/LP variants per individual. Bars represent the number of participants carrying one, two, three, or four incidental P/LP variants after excluding NBS-associated and ACMG SF genes. Numbers above bars indicate participant counts.

After excluding NBS-associated and ACMG SF genes, 61/118 participants (51.7%) harbored at least one reportable incidental finding: 35 (29.7%) carried one P/LP variant, 19 (16.1%) carried two, 4 (3.4%) carried three, and 3 (2.5%) carried four, for a total of 97 reportable incidental variants (Figure 1).

### 3.8. Carrier Status

**Figure 2:**
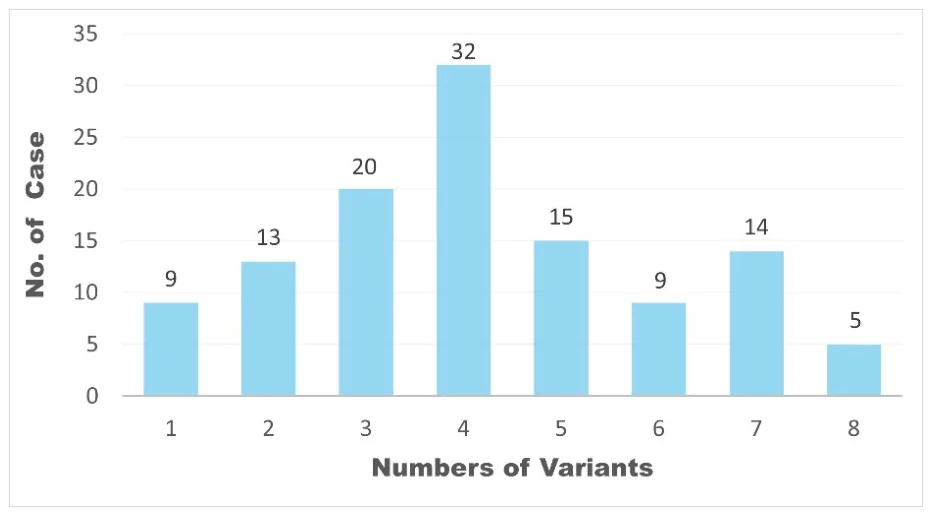
Number of carrier-status variants reported per individual. Each bar represents the number of participants with a given count of carrier-status autosomal-recessive variants identified across the exome. Mean 4.15 variants per individual (median 4; range: 1–8).

Exome-wide carrier status for at least one autosomal-recessive disease was identified in 117/118 participants (99.2%) (Figure 2), with a mean of 4.15 carrier variants per individual (median 4; range 1–8). The top five carrier genes were *GJB2* (24/118, 20.3%), *CD36* (9/118, 7.6%), *DUOX2* (9/118, 7.6%), *NDUFAF8* (6/118, 5.1%), and *PAH* (6/118, 5.1%).

## 4. Discussion

This retrospective study describes WES outcomes in 118 Taiwanese neonates referred following abnormal biochemical NBS—one of the first systematic analyses of second-tier neonatal WES in a Taiwanese cohort. The 80.5% combined diagnostic-plus-carrier yield substantially improves clinical interpretability beyond biochemical results alone, and benchmarks favorably against contemporary international second-tier cohorts [15,29].

### 4.1. Diagnostic Yield and Clinical Utility

The confirmed-diagnosis rate of 42.4% and carrier detection rate of 38.1% are broadly consistent with previously published second-tier WES cohorts [30,31,32]. Importantly, WES enabled clinically critical disambiguations: (i) citrullinemia type I versus type II, conditions with distinct prognoses and management strategies; (ii) Pompe disease versus *GAA* pseudodeficiency, the predominant cause of biochemical false positives in East Asians [5,23]; and (iii) classical Fabry disease versus the IVS4+919G>A cardiac variant, which has a fundamentally different natural history and surveillance requirement. For Fabry disease, the predominance of IVS4+919G>A (24/25 confirmed males) mirrors its well-documented overrepresentation in Taiwanese and East Asian populations [6,21].

The 19.5% unresolved rate highlights limitations of WES for structural variants, large indels, tandem repeats, and regions of reduced coverage—particularly relevant for *CBS*, *PAH*, and *SLC25A13*. Integration of complementary technologies—long-read sequencing, optical genome mapping, and RNA sequencing—is expected to narrow this diagnostic gap. However, no single genomic modality currently satisfies all primary NBS requirements, and a tiered approach combining biochemistry and genomics remains optimal [33]. For patients who remained undiagnosed, biochemical metabolic profiles and clinical manifestations will be monitored longitudinally. When newly identified abnormalities are suggestive of the originally suspected disorder, the WES data will be reanalyzed, and, when appropriate, third-generation long-read sequencing may be performed to further investigate the underlying genetic etiology.

### 4.2. Secondary and Incidental Findings Under ACMG SF v3.3

The 4.2% SF rate in our cohort is consistent with European estimates (3.4–4.5%) and 1.2% in African populations [34]. A Taiwan-specific study by Kuo et al. reported actionable SFs at a comparable frequency among Taiwanese exomes [26]. The identified genes (*TTN*, *LDLR*, *PTEN*, *RYR1*, *TP53*) are all associated with conditions for which early surveillance or intervention can meaningfully alter outcomes. Although ACMG SF v3.3 (84 genes, 2025) newly adds *ABCD1* and further cardiovascular genes, X-ALD is a primary referral category in the Taiwanese NBS program; the two X-ALD-confirmed neonates and two carriers in our cohort were therefore reported as primary findings rather than secondary findings. Their identification nonetheless underscores the clinical importance of *ABCD1*, as cerebral ALD is potentially preventable through pre-symptomatic hematopoietic stem-cell transplantation [35].

The 51.7% incidental-finding prevalence substantially exceeds the 3.4% reported for high-penetrance actionable variants in population cohorts [34]. The discrepancy reflects allelic heterogeneity, complex inheritance patterns, and the inclusion of variants associated with late-onset conditions, incomplete penetrance, or mild phenotypes. For example, *FLG* variants (*n* = 21) confer graded risk for ichthyosis vulgaris and atopic dermatitis through semi-dominant inheritance rather than deterministic disease [36]. Clinicians must communicate this nuance clearly to families, distinguishing genetic risk from diagnostic certainty.

### 4.3. Exome-Wide Carrier Burden and the East Asian Genomic Architecture

The near-universal carrier burden (99.2% with at least one recessive carrier variant; mean 4.15 variants) is consistent with prior exome-wide carrier analyses and reflects the large number of autosomal-recessive disease genes across the human genome [37]. The high *GJB2* carrier frequency (20.3%) is consistent with published East Asian data and is clinically relevant for hearing-screening follow-up counseling [38]. A recent population-scale Chinese genomic NBS cohort independently reported a high frequency of actionable SFs and a broad recessive-carrier landscape in East Asian newborns [39].

### 4.4. Implications for Pediatric Genomic Newborn Screening Program Design

Our results, viewed alongside BabyScreen+ [11], Early Check [12], and the GUARDIAN study [13], collectively support a tiered NBS architecture: (1) biochemical first-tier screening optimized for common Mendelian IEMs, followed by (2) genomic second-tier testing triggered by biochemical abnormality, with population-contextualized gene panels and curated hotspot/pseudodeficiency annotations. These two independent methods increase sensitivity and specificity of the overall test. This tiered approach is particularly critical in pediatric care settings where timely diagnosis directly informs treatment initiation.

### 4.5. Ethical Considerations

Return of secondary, incidental, and carrier findings to families of neonates raises complex ethical questions, balancing the child’s right to an open future against parental interests in genetic information [40,41]. Our program’s approach—comprehensive pre-test counseling and parental choice to receive or decline each category of findings—aligns with the NC NEXUS project’s finding that >90% of parents elect to receive at least one category of additional genomic information [10], and with recent BabyScreen+ qualitative data showing that parents value receiving results when supported by a robust counseling pathway [42].

### 4.6. Limitations

Several limitations should be acknowledged. First, as a retrospective single-center study with 118 participants, statistical power is limited and selection bias cannot be excluded. Second, phasing could not be established from WES alone; participants with two reportable variants in the same recessive gene were classified as genomic positives, acknowledging the possibility that both variants reside in cis. Third, WES has well-characterized limitations for structural variants, large indels, tandem repeats, and CNVs, contributing to the 19.5% unresolved rate. Fourth, longitudinal clinical follow-up was unavailable to validate phenotypic outcomes for incidental/secondary findings. Finally, parental genotyping was incomplete, limiting phase determination and de novo verification.

## 5. Conclusions

Second-tier whole exome sequencing following abnormal biochemical NBS provides substantial diagnostic value in Taiwanese neonates, achieving a 42.4% confirmed-diagnosis rate and identifying carriers in an additional 38.1%. The 4.2% secondary-finding rate (*TTN*, *LDLR*, *PTEN*, *RYR1*, *TP53*), reconfirmed under ACMG SF v3.3—51.7% incidental-finding prevalence, and near-universal carrier burden (99.2%) collectively highlight the complexity of neonatal genomic reporting and the indispensable role of pre- and post-test genetic counseling. Taiwan-specific allele frequencies—particularly cardiac-type Fabry *GLA* IVS4+919G>A, *GAA* pseudodeficiency, and *GJB2* carrier prevalence—underscore the importance of population-contextualized interpretation for pediatric patients. These findings support the complementary role of WES alongside biochemical NBS rather than as a replacement and provide a reference dataset to guide counseling practices and variant interpretation for East Asian neonatal genomic programs. Prospective multicenter studies with larger cohorts, trio sequencing, and long-term phenotypic follow-up are warranted to further define the clinical utility and optimal implementation of genomic newborn screening in Taiwan.

## Figures and Tables

**Table 1:** WES outcomes by disorder category in 118 Taiwanese neonates with abnormal newborn screening.

Disorder Category	Carrier	Confirmed	Unresolved	Total
Amino Acid Disorders	21	10	7	38
Citrullinemia (type I/II)	21	3	0	24
Phenylketonuria	0	7	0	7
Homocystinuria	0	0	7	7
Fatty Acid Oxidation Disorders	3	8	2	13
Adrenoleukodystrophy (X-ALD)	2	2	1	5
CPT II Deficiency	1	0	0	1
Carnitine Transporter Defect	0	3	0	3
Glutaric Aciduria Type I	0	3	0	3
VLCADD	0	0	1	1
Organic Acid Disorders	3	1	2	6
3-MCC Deficiency	3	1	0	4
Methylmalonic Acidemia	0	0	1	1
Isovaleric Acidemia	0	0	1	1
Lysosomal Storage Diseases	18	31	12	61
Fabry Disease	0	25	11	36
Pompe Disease	18	6	1	25
Total	45	50	23	118

3-MCC, 3-methylcrotonyl-CoA carboxylase; CPT II, carnitine palmitoyl transferase II; VLCADD, very long-chain acyl-CoA dehydrogenase deficiency; X-ALD, X-linked adrenoleukodystrophy. “Unresolved” indicates no P/LP variant detected by WES.

**Table 2:** Secondary findings detected under ACMG SF v3.2 criteria and reconfirmed under v3.3.

Gene	Patient ID	Variant (HGVS)	Associated Condition	TWB (per 10 k)	Class.
*TTN*	ED548-1	c.1800+1G>A (p.?)	Dilated cardiomyopathy	20	P/LP
*LDLR*	ED357-1	c.268G>A (p.Asp90Asn)	Familial hypercholesterolemia	25	LP
*PTEN*	ED051-1	c.2T>A (p.0?)	PTEN hamartoma tumor syndrome	N/A	P/LP
*RYR1*	ED102-1	c.2455C>T (p.Arg819X)	Malignant hyperthermia susceptibility	0	P/LP
*TP53*	ED780-1	c.514G>A (p.Val172Ile)	Li-Fraumeni syndrome	5	LP

TWB, Taiwan Biobank; P, pathogenic; LP, likely pathogenic. Variant frequency expressed per 10,000 individuals. All variants remained reportable under ACMG SF v3.3 (2025).

## Data Availability

The data presented in this study are available on request from the corresponding author. The data are not publicly available due to privacy.

## Abbreviations

The following abbreviations are used in this manuscript:

ACMG	American College of Medical Genetics and Genomics
ALD	Adrenoleukodystrophy
AMP	Association for Molecular Pathology
CNV	Copy number variant
HPO	Human Phenotype Ontology
IEM	Inborn error of metabolism
LSD	Lysosomal storage disease
MS/MS	Tandem mass spectrometry
NBS	Newborn screening
NGS	Next-generation sequencing
P/LP	Pathogenic/likely pathogenic
PKU	Phenylketonuria
SF	Secondary finding
TVGH	Taipei Veterans General Hospital
VUS	Variant of uncertain significance
WES	Whole exome sequencing
WGS	Whole genome sequencing
